# Effects of a Brief Qigong-based Stress Reduction Program (BQSRP) in a distressed Korean population: a randomized trial

**DOI:** 10.1186/1472-6882-13-113

**Published:** 2013-05-25

**Authors:** Eun-Young Hwang, Sun-Yong Chung, Jae-Heung Cho, Mi-Yeon Song, Sehyun Kim, Jong-Woo Kim

**Affiliations:** 1 Departments of Korean Neuropsychiatry, College of Korean Medicine and Institute of Korean Medicine, Kyung Hee University, 1 Hoegi-dong, Dongdaemun-gu, Seoul 130-701, Republic of Korea; 2 Department of Korean Rehabilitation, College of Korean Medicine and Institute of Korean Medicine, Kyung Hee University, 1 Hoegi-dong, Dongdaemun-gu, Seoul 130-701, Republic of Korea; 3 Graduate School of Dankook University Jukjeon Campus, 152 Jukjeon-ro, Suji-gu, Yongin-si, Gyeonggi-do 448-701, Republic of Korea

## Abstract

**Background:**

Distressed individuals in Korea may benefit from the practice of mind–body exercises such as Qigong. However, the effectiveness of such techniques needs to be investigated.

**Methods:**

Fifty participants who were eligible to this study were randomized into a group receiving a 4-week intervention of a brief Qigong-based stress reduction program (BQSRP) or a wait-list control group. Before and after the intervention period, saliva samples were collected and questionnaires were completed on perceived stress, anxiety, “*Hwa-Byung*” (anger syndrome), and quality of life. Salivary cortisol has emerged in mind-body therapy research as an easy-to-collect, relatively inexpensive, biologic marker of stress. Salivary corisol were collected to evaluate physiological effect of BQSRP. Between-group comparisons of change from baseline to study completion were analyzed by analysis of covariance for the Perceived Stress Scale and independent two sample *t-*tests for other measures.

**Results:**

Compared with the control group, the BQSRP intervention group displayed significantly larger decreases in Perceived Stress Scale scores (*p* = 0.0006), State Anxiety scores (*p* = 0.0028), Trait Anxiety scores (*p <* 0.0001), personality subscale scores of the *Hwa-Byung* Scale (*p* = 0.0321), symptoms scores of the *Hwa-Byung* Scale (*p* = 0.0196), and a significantly larger increase in World Health Organization Quality of Life Abbreviated version scores (*p*s < .05). Salivary cortisol levels were not changed.

**Conclusions:**

The BQSRP appears to be effective in reducing stress perception, anxiety, anger, and improving quality of life (KCT0000056).

## Background

Mental stress is a serious problem in today’s society. Failure to cope with stress can have adverse effects that include increased incidences of anxiety and mood disorders as well as physical disorders [1]. In South Korea, one study classified 73% of employees as a potential-stress group, 22% of employees as a high-stress group, and only 5% of employees as a non-stress group [2].

Several recent studies have demonstrated the stress relieving benefits of mind–body therapy, such as progressive muscle relaxation, yoga [3], autogenic training (AT) [4], Qigong [5], and meditation [6]. AT is effective for anxiety relief, mild to moderate depression, functional sleep disorders, mild to moderate hypertension, tension-type headache, migraine, coronary heart disease, asthma, Raynaud’s disease, and somatoform pain disorder (unspecified type) [4]. A mindfulness-based stress reduction (MBSR) program can improve coping for individuals suffering from cancer and chronic pain, anxiety, depression, and stress [6]. Qigong is an ancient Chinese mind control exercise involving breathing methods and “vital energy”, which has a positive effect on health and allows the exerciser to gain control over Qi, the life-energy that flows in channels (meridians) in the body [5,7]. Qigong has been reported to be effective for depression, anxiety, perceived stress level, hypertension, fibromyalgia, pain, immune function, lipid metabolism, and hormonal variation in the sympathetic nervous system and/or hypothalamic-pituitary-adrenal (HPA) axis [5,8].

Cortisol is the major stress hormone in the human organism related to HPA axis and there were evidences by decreased cortisol levels following mind-body stress reduction interventions [9].

Most common mind–body interventions require a considerable commitment of time and money to complete the training. The MBSR program requires 8–10 weeks, and consists of 2–2.5-hour weekly sessions, one all-day intensive session, and daily practice [10].

Because even a short 4–6-week program of mind–body therapy may be effective for stress management [11-13], the authors developed a simple, 4-week 15-minute-daily stress reduction program that is easy to practice and contains the basic components of Qigong.

This study was a preliminary investigation to evaluate the effectiveness of this brief Qigong-based stress reduction program (BQSRP) for alleviating subjective stress and improving quality of life in distressed subjects.

## Methods

### Participants

Participants were recruited from the local community by newsletters and advertisements posted in newspapers in Seoul, Korea, commencing in September 2009. Participants were included if they were 20 to 60 years old and if they had symptoms of stress at least regularly or often and if they felt they did not have control over their everyday lives because of that stress.

Among those included, subjects were excluded if they had experience with another mind–body practice (e.g., yoga or meditation) for at least 1 month in the previous year. They were also excluded if they had taken medicine or non-pharmacological therapy related to a psychological disorder or heart disease or had taken steroid medication in the past month. Participants with suicidal ideation, psychotic symptoms such as delusions or hallucinations, or had an insufficient understanding of the Korean language were also excluded. Participants who completed the study received compensation for their efforts.

**Figure 1:**
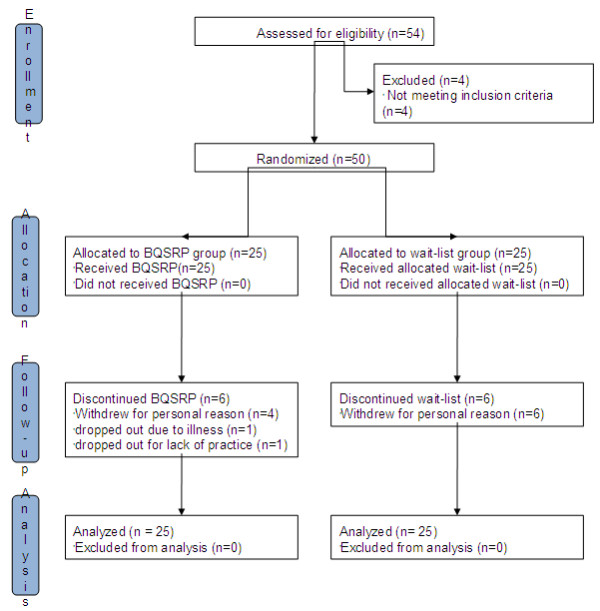
Flow diagram of the study.

The 50 participants were numbered sequentially according to their enrollment order by one of the authors (EYH). They were randomized in an intervention group that received the BQSRP (*n* = 25) or a wait-list control group (*n* = 25) that received the BQSRP after the intervention group finished their training (Figure 1). Random allocation of the BQSRP participants was performed using Random Allocation Software version 1.0.0 (http://www.saghaei.net) by one of the authors (SYC). We used the stratified randomization by matching gender.

The study protocol was approved in advance by the institutional review board of the Kyung Hee University Hospital at Gangdong in Seoul, Korea. Written informed consent was obtained from all participants after the study had been completely and clearly explained. Data were collected at the same institution.

### Outcome measures

#### Psychological outcomes

##### Perceived Stress Scale (PSS)

The PSS assesses the degree to which situations in one’s life are appraised as stressful [14]. The 10 items elicit information about feelings and thoughts during the past month and how often the respondent felt a certain way in a specific situation. The scoring system uses a five-point Likert scale with a maximum stress score of 40. The PSS has an internal consistency (Cronbach’s α) coefficient of 0.85 and well-established validity [15]. Scores were imputed for participants missing one PSS item. Scores for these missing items were computed by calculating the mean score for the remaining nine of the 10 items. This was the primary outcome measure.

### State-Trait Anxiety Inventory (STAI*)*

Anxiety was measured with the Korean version of the STAI, a widely-used measure with well-established validity and reliability [16]. It comprises two 20-item self-report subscales containing words descriptive of anxiety that are rated on a four-point Likert scale. Items on the state anxiety subscale deal with how the person is feeling “right now, at this moment”. Items on the trait anxiety subscale measure how the respondent is “generally feels”. The STAI scores were only calculated if at least 17 items (out of 20) were answered. For three or fewer missing items, a value of 2.0 was substituted.

### *Hwa-byung* scale

“*Hwa-Byung*” is a Korean culture-bound syndrome that translates into English as “anger syndrome”. It is included in Culture-Bound Syndromes described in Appendix I in the Diagnostic and Statistical Manual of Mental Disorders (4th edition; DSM-IV). The syndrome is caused by suppression of anger and results in physical symptoms of chest discomfort and heartburn as well as feelings of humiliation and shame [17]. The *Hwa-Byung* personality subscale comprises 16 items, and the *Hwa-Byung* symptoms subscale comprises 15 items, which are scored using a five-point Likert scale. The personality subscale assesses the degree to which suppression of emotion, interpersonal characteristics, stubbornness, and coping methods contribute to *Hwa-Byung*. The symptoms subscale assesses the psychological and physical symptoms of *Hwa-Byung*. The internal consistency (Cronbach’s α) coefficient of the *Hwa-Byung* Scale has been reported as 0.92, with the *Hwa-Byung* personality subscale at 0.85 and *Hwa-Byung* symptoms subscale at 0.91 [18]. The subscales were only calculated if fewer than two items were missing from each subscale, in which case the mean of the other items in the subscale was substituted.

### World Health Organization Quality of Life Abbreviated version (WHOQOL-BREF)

The WHOQOL-BREF is a shortened version of the WHOQOL-100 QOL (Quality of Life questionnaire). In this study, the Korean version of the WHOQOL-BREF was used to obtain the degree of contentment in four domains of QOL: physical health, psychological health, social relationships, and environment [19]. The WHOQOL-BREF comprises 26 items and has been reported to show satisfactory internal consistency (Cronbach’s α ranging from 0.58 for the social domain to 0.77 for physical health). It also has adequate test-retest reliability (0.732–0.799) and discriminant validity. Where an item is missing, the mean of other items in the domain can be substituted. When more than two items are missing from the domain, the domain score should not be calculated, except for domain 3 in which more than one missing item is required to cancel the calculation.

### Physiological outcomes

#### Salivary cortisol

Saliva samples were collected using Salivettes (Salimetrics, State College, PA, USA). Salivary cortisol exhibits a diurnal rhythm and increases rapidly within 60 minutes after awakening in people experiencing high stress [20]. In the present study, saliva was collected 1 hour after awakening, between 7 and 9 a.m. No eating and no drinking except for water was allowed prior to saliva collection. The collected Salivettes were kept cold using icepacks during delivery to the hospital and were kept frozen at −20°C until assayed. Saliva samples were thawed and centrifuged at 3000 rpm for 15 minutes at room temperature. An enzyme-linked immunosorbent assay kit (Salimetrics) was used to determine cortisol levels. The expected range of salivary cortisol of an adult in the morning is 0.094–1.551 [21].

### Study interventions

#### Procedure

All participants were randomized into the intervention or wait-list control group. At baseline, all participants completed the questionnaire at the hospital to assess psychological well-being, and the intervention group started the BQSRP training. After 4 weeks, at the end of the training period, all participants were asked to complete the same questionnaires. The control group had no intervention for 4 weeks, but was asked during a telephone interview with the trainer to describe any stressful events and state of health every week. After 4 weeks, the control group was given the BQSRP training.

Salivettes were distributed after completing the consent form, and saliva samples were collected at both baseline and after 4 weeks (Figure 1).

### BQSRP

The training consisted of four weekly group sessions at the hospital, with a 2-hour session during week 1 and 1-hour sessions thereafter, as well as brief Qigong meditation (BQM) home practice twice daily for the instructed length of time. During the four weekly sessions, material related to relaxation, meditation, and mind–body connections and BQM instructions were given. Session groups of six to 10 participants were led by one of the authors (JWK) who has 20 years of Qigong training. BQM in this program took 15 minutes and consisted of three different techniques: 1) relaxed breathing, 2) relaxing the body, and 3) gathering Qi and self-healing. Relaxed breathing consisted of deep respiration with longer exhalation and abdominal breathing while counting down from 10. This was repeated three times. Relaxing the body involved passive concentration on bodily perceptions, especially focusing on feeling the weight and warmth of both hands. Gathering Qi and self-healing consisted of sensing Qi sensations and gathering Qi between both hands. Participants were also given a booklet, an MP3 player containing an audio BQM guide to aid them in their daily practice, and a meditation log in which they recorded the actual time spent in daily meditation and which they later handed in. Participants were required to engage in at least 60% of the daily practice recommended.

### Sample size

Previous research examining the effectiveness of Qigong training on reducing PSS scores using a wait list as the control [13] revealed that at a significance level of 0.05 and desired power of 0.80, 21 people per group would be required to detect a significant difference between the intervention and control groups. Furthermore, we considered that, given a drop-out rate of 20%, 25 persons were needed per group.

### Statistical analyses

All analysis was done by Intent-to-Treat (i.e., as randomized) principle. Paired *t*-test was done for within group comparisons. Between-group comparisons of demographic characteristics, baseline measures, and changes in measures from baseline to study completion were analyzed by two-sample independent *t-*tests. If baseline measures were different, we used an analysis of covariance (ANCOVA) to control for baseline when comparing the two groups. All reported *P*-values were based on two-sided tests, and a *P*-value < 0.05 was considered significant. All analyses were conducted with SAS for Windows (version 9.1, SAS Institute, Cary, North Carolina, USA).

## Results

### Subject characteristics

It took about one month to recruit for participants. Fifty-four subjects were recruited and four of them were excluded because they did not meet the inclusion criteria.

Fifty subjects (39 females and 11 males) were matched for gender and then randomized (25 in each treatment group) by computer. Twelve subjects (six from the BQSRP group and six from the wait-list group) did not complete the study. Four participants from the BQSRP group withdrew for personal reasons, one participant dropped out as a result of acute illness necessitating an operation, and one participant discontinued because of lack of practice. Six subjects from the wait-list group withdrew for personal reasons and were lost to follow-up. Subjects ranged from 21–59 years of age and had 12–18 years of education. One participant from the BQSRP group refused to complete the baseline questionnaires and did not continue the study, one participant in the BQSRP group did not complete the STAI and WHOQOL-BREF at baseline, and one participant in the wait-list group did not complete the *Hwa-Byung* symptoms subscale. The cortisol level of three samples (two at baseline and one at the end point) was very low compared with the expected value, and one sample at study completion could not be assessed because of the small amount of the sample. Therefore, four samples (two from the BQSRP group and two from the control group) were excluded from analysis.

Cronbach’s alphas were 0.87 for the PSS, 0.94 for the state anxiety subscale, 0.91 for the trait anxiety subscale, 0.80 for the *Hwa-Byung* personality subscale, and 0.93 for the *Hwa-Byung* symptoms subscale. The WHOQOL-BREF ranged from 0.64 for social relationships to 0.83 for environment.

Baseline characteristics are presented in Additional file 1. There were no significant differences between groups in demographic characteristics such as age, gender, and education level. The baseline scores for the PSS in the BQSRP group were significantly higher than those in the control group. However, the baseline scores for the STAI, *Hwa-Byung* personality subscale, *Hwa-Byung* symptoms subscale, and WHOQOL-BREF were not significantly different between the two groups.

### Psychological outcomes

The BQSRP intervention produced significant decreases in perceived stress, state and trait anxiety, *Hwa-Byung* personality, and WHOQOL-BREF scores except for social relationships within the BQSRP group (Additional file 1). With the exception of trait anxiety, there were no significant changes in the wait-list group (Additional file 1).

The BQSRP group reported significant decreases in perceived stress, state and trait anxiety, *Hwa-Byung* personality, and *Hwa-Byung* symptoms compared with the control group (Additional file 1). Scores for perceived stress, the STAI, and the *Hwa-Byung* Scale indicated the BQSRP group had experienced clinical improvements in stress relief at 4 weeks. Contrarily, all scores on the WHOQOL-BREF subscales increased in the BQSRP group compared with the wait-list group, with statistically significant differences between groups (Additional file 1).

### Physiological outcomes

There were no significant differences between groups in salivary cortisol level at baseline and at the end of the BQSRP intervention (Additional file 1). There were no significant differences by gender or age.

## Discussion

This study was a preliminary investigation to determine whether a stress reduction program based on the mind–body intervention of Qigong could be beneficial for patients with stress-related problems. The findings revealed that even brief Qigong training as a stress-reduction technique in simple meditation practice was associated with improvements in subjective stress and psychological well-being in a general sample of distressed adults. The BQSRP group demonstrated highly significant decreases in all three stress outcome measures (PSS, STAI, and *Hwa-Byung* Scale) and increased WHOQOL-BREF.

The PSS was reported as 19.7 after stressful events within healthcare workers in Korea, which is similar to the baseline score of our participants [22]. In the United States of America, the PSS was reported as 14.1 in the minority group of general population [15]. Therefore, the change of PSS from 20.7 to 15.4 in this study was considered clinical significant.

The state anxiety was reported as 42.97 in male and 41.96 in female and the trait anxiety was reported as 44.26 in male and 44.85 in female among the university students [16]. The changes of State anxiety from 47.1 to 38.9 and of Trait anxiety from 48.1 to 41.0 in this study were considered clinically significant.

In the WHOQOL-BREF of this study, all subscale scores of baseline were below mean scores in general population [19]. 4 weeks after, all scores increased above mean scores in general population. Therefore, the changes of WHOQOL-BREF subscales were considered clinical significant.

Lack of money and time can limit available leisure time and are frequently reported barriers to individuals staying physically active [23]. Most common mind–body interventions require more than 8 weeks to be completed [9], which is a deterrent to participation for many individuals. Previous research has shown that even a short-term program may be effective for stress management [10]. The present results reinforce this view. Therefore, the BQSRP may be beneficial for distressed individuals.

The changes in WHOQOL-BREF subscale scores over time were significantly different between the two groups. Cronbach’s alphas for WHOQOL-BREF ranged from 0.63 to 0.83, these coefficients were similar to those in standardization or validation studies and support the reliability of the present results. Negative perception of stress factors can cause stress responses and lead to mental and physical disorders. A previous paper reported that meditation reduces negative and increases positive emotions [24]. Even though there was no change in life events, reported quality of life improved in the study participants, indicating that this program caused changes in stress perception.

*Hwa-Byung* is a chronic anger syndrome, in which anger is thought to be chronically suppressed, “accumulates and becomes dense”, and is characterized by unique symptoms including partially suppressed subjective anger and somatic and behavioral manifestations of that anger, different from depressive syndrome in terms of its symptom profile [25]. A previous study reported that *Hwa-Byung* patients had various comorbidities including major depressive disorder (60.7%) and generalized anxiety disorder (16.9%), but there were patients who had only *Hwa-Byung* without comorbidity, and the distribution of single diagnoses and comorbid diagnoses was similar for *Hwa-Byung*, major depressive disorder, and generalized anxiety disorder [26]. The personality subscale score on the *Hwa-Byung* Scale will be high if someone is suppressing their emotions, is passively withdrawn, is self-critical, and is perseverant [18]. In the BQSRP participants, the personality subscale score on the *Hwa-Byung* Scale was somewhat reduced. The mean change in that subscale score was only 7%, about half that of the *Hwa-Byung* symptoms subscale score, but was sufficient to elicit behavioral changes. The effectiveness of mind–body relaxation programs for *Hwa-Byung* patients has been reported [27], and the present study produced a similar result. Therefore, mind–body therapy may be effective for *Hwa-Byung* patients.

In this study, the BQSRP-trained participants reported a decrease in distress and an increase in quality of life. However, salivary cortisol levels did not change compared with baseline. It should be noted that all our subjects’ salivary cortisol levels were within the low-normal range for healthy older adults. Heterogeneity in age profiles may have attenuated the cortisol results for several possible reasons. Some people do not show typical diurnal cortisol secretion rhythm [28]. Furthermore, the PSS results may not correlate with perceived stress level and cortisol secretion [29], and it is debatable whether different relaxation techniques such as mindfulness meditation affect salivary cortisol levels [30]. As such, it appears that there was little opportunity for salivary cortisol values to be influenced by BQSRP training over the 4-week period. The salivary cortisol assay is non-invasive, making multiple sampling easy and stress-free. However, the testing parameters, saliva collection time, and heterogeneity of the group may have affected the results. There may have been a true lack of physiologic effect of this BQSRP intervention or a need for more sensitive measures of stress. Other markers such as salivary IgA or amylase may be more sensitive to the relaxation response [31]. In addition, the primary measure of this study was the perceived stress scores (PSS), so the sample size might have been inadequate to find out the change on the cortisol level.

### Limitations

A limitation of this study was the restriction of results to the immediate post-intervention period. As this study was a wait-list control, the contact with the trainer might have influenced the outcomes. The wait-list control subjects were trained for 4 weeks, the same as the Qigong group. Therefore, the authors could not assess the difference between the two groups over time. Investigation of long-term outcomes is needed. In addition, salivary cortisol has a circadian rhythm and is ideally sampled more than twice a day at different time across 2–3 days. Because of a limited budget, it was sampled only once in this study. Therefore, further study is warranted.

## Conclusions

It is plausible that a 4-week BQSRP intervention is effective in reducing stress perception, anxiety, anger, and improving quality of life. Such a program may have advantages over more established programs in many situations, particularly in regard to length and cost of training.

## Competing interests

No competing financial interests exist.

## Authors’ contributions

EYH, SYC, JWK and MYS contributed to the conception and design of the study. JWK performed the BQSRP, EYH and JHC acquired the data. SHK performed the statistical analysis. EYH and SYC drafted the manuscript and all authors contributed to further writing of the manuscript. All authors read and approved the final manuscript.

## Pre-publication history

The pre-publication history for this paper can be accessed here:

http://www.biomedcentral.com/1472-6882/13/113/prepub

## Supplementary Material

Additional file 1: Table S1 Demographic and psychological characteristics and quality of life of study participants. **Table S2**. PSS, STAI, *Hwa-Byung* Scale, and WHOQOL-BREF scores within groups. **Table S3**. Changes in PSS, STAI, Hwa-Byung, and WHOQOL-BREF scores from baseline. **Table S4**. Comparison of salivary cortisol hormone levels at baseline and change from baseline between groups (μg/dl). Click here for file
