# Supplementary material for: Effects of a Brief Qigong-based Stress Reduction Program (BQSRP) in a distressed Korean population: a randomized trial
Source: BMC Complement Altern Med. 2013 May 25;13:113. doi: 10.1186/1472-6882-13-113 (PMC3680074; doi:10.1186/1472-6882-13-113)
Supplement: Additional file 1: Table S1 — Demographic and psychological characteristics and quality of life of study participants. Table S2. PSS, STAI, Hwa-Byung Scale, and WHOQOL-BREF scores within groups. Table S3. Changes in PSS, STAI, Hwa-Byung, and WHOQOL-BREF scores from baseline. Table S4. Comparison of salivary cortisol hormone levels at baseline and change from baseline between groups (μg/dl). [file 1472-6882-13-113-S1.docx]

# Tables

| Table 1. Demographic and psychological characteristics and quality of life of study participants. | | | | | |
| --- | --- | --- | --- | --- | --- |
|  | **BQSRP**  **(n=25)** | | **Wait list**  **(n=25)** | | ***P*** |
| Age, years | 41.48 | ±11.57 | 40.56 | ±11.75 | 0.7815 |
| Years of Education | 15.25 | ±2.19 | 15.12 | ±2.01 | 0.8294 |
| PSS ^†^ | 20.71 | ±5.88 | 17.72 | ±4.17 | 0.0450* |
| State anxiety^‡^ | 47.09 | ±9.98 | 45.04 | ±11.72 | 0.5197 |
| Trait anxiety^‡^ | 48.13 | ±10.22 | 45.60 | ±8.78 | 0.3611 |
| *Hwa-Byung* personality^†^ | 33.13 | ±7.55 | 30.24 | ±5.95 | 0.1432 |
| *Hwa-Byung* symptoms^†§^ | 22.67 | ±11.70 | 19.50 | ±7.87 | 0.2771 |
| WHOQOL-BREF^‡^ |  |  |  |  |  |
| Physical QOL | 12.75 | ±2.42 | 12.37 | ±3.29 | 0.6534 |
| Psychological QOL | 11.30 | ±2.50 | 11.39 | ±2.72 | 0.9138 |
| Social Relationships | 12.12 | ±2.34 | 12.64 | ±2.22 | 0.4296 |
| Environment | 11.50 | ±2.31 | 11.90 | ±2.50 | 0.5683 |
| Data indicate mean ± standard deviation. * *P* < 0.05, p-values obtained by two-tailed independent two-sample t-tests.^†^ The sample size of BQSRP is 24 because one participant in BQSRP group refused to complete the baseline questionnaires and did not continue the study.^‡^ The sample size of BQSRP is 23 because one participant in BQSRP group did not complete the questionnaires for STAI and WHOQOL-BREF at baseline. ^§^The sample size of waiting is 24 because one participant in waiting group did not complete the questionnaire for *Hwa-Byung* symptom. | | | | | |

## Table 2. PSS, STAI, *Hwa-Byung* Scale, and WHOQOL-BREF scores within groups.

|  | | Group | Baseline | 4 weeks | Change | 95% CL  (Lower CL; Upper CL) | ***P*** |
| --- | --- | --- | --- | --- | --- | --- | --- |
| PSS | | B | 20.7±5.9 | 15.4±3.8 | -4.3±4.7 | (-6.5; -2.0) | 0.0010^**^ |
|  | | W | 17.7±4.2 | 19.2±4.2 | 0.8±3.3 | (-0.7; 2.4) | 0.2765 |
| State anxiety^‡^ | | B | 47.1±9.9 | 38.9±10.0 | -7.8±9.5 | (-12.5; -3.1) | 0.0028^**^ |
|  | | W | 45.0±11.7 | 48.7±10.6 | 1.8±8.6 | (-2.4; 5.9) | 0.3774 |
| Trait anxiety^‡^ | | B | 48.1±10.2 | 41.0±7.7 | -6.4±6.5 | (-9.6; -3.2) | 0.0006^**^ |
|  | | W | 45.6±8.8 | 49.8±9.4 | 3.4±4.3 | (1.3; 5.5) | 0.0029^**^ |
| *Hwa-Byung* personality | | B | 33.1±7.5 | 30.7±6.8 | -2.4±4.4 | (-4.5; -0.3) | 0.0298^*^ |
|  |  | W | 30.2±6.0 | 32.9±6.4 | 1.2±5.3 | (-1.4; 3.7) | 0.3558 |
| *Hwa-Byung* symptoms^§^ | | B | 22.7±11.7 | 18.0±6.5 | -2.8±7.1 | (-6.3; 0.6) | 0.0969 |
|  |  | W | 19.5±7.9 | 22.7±9.0 | 2.5±6.1 | (-0.6; 5.6) | 0.1026 |
| WHOQOL-BREF^‡^ | |  |  |  |  |  |  |
| Physical QOL | | B | 12.7±2.4 | 14.3±1.9 | 1.6±2.4 | (0.4; 2.8) | 0.0109^*^ |
|  | | W | 12.4±3.3 | 11.7±3.8 | -0.5±2.7 | (-1.9; 0.8) | 0.3966 |
| Psychological QOL | | B | 11.3±2.5 | 12.7±2.3 | 1.5±1.9 | (0.6; 2.5) | 0.0038^**^ |
|  |  | W | 11.4±2.7 | 11.3±2.7 | 0±1.5 | (-0.7; 0.7) | 1.0000 |
| Social Relationships | | B | 12.1±2.3 | 13.1±1.8 | 1.0±2.5 | (-0.3; 2.2) | 0.1143 |
|  |  | W | 12.6±2.2 | 11.6±2.8 | -0.9±1.9 | (-1.8; 0.0) | 0.0552 |
| Environment | | B | 11.5±2.3 | 12.7±2.6 | 1.4±1.7 | (0.6; 2.3) | 0.0024^**^ |
|  | | W | 11.9±2.5 | 12.0±2.6 | 0.1±1.8 | (-0.8; 1.0) | 0.7578 |
| Data indicate mean ± standard deviation. ** p < 0.01, * p < 0.05, p-values obtained by two-tailed paired t-tests within groups. B : BQSRP group, W : Wait list group  Baseline : B (n=24), W (n=25)  4weeks : B (n=19), W (n=19)  Change : B (n=19), W (n=19) ^‡^ The baseline sample size of BQSRP is 23 because one participant in BQSRP group did not complete the questionnaires for STAI and WHOQOL-BREF and the change sample size of BQSRP is 18.^§^The baseline sample size of waiting is 24 because one participant in waiting group did not complete the questionnaire for *Hwa-Byung* symptom and the change sample size of waiting is 18. | | | | | | | |
|  |  | | | | | | |

## Table 3. Changes in PSS, STAI, Hwa-Byung, and WHOQOL-BREF scores from baseline.

|  | **BQSRP (n=19)** | | **Waiting (n=19)** | |  | ***P*** |
| --- | --- | --- | --- | --- | --- | --- |
| PSS^†^ | -4.26 ± 4.72 | | 0.84 ± 3.27 | |  | 0.0006^**^ |
| State anxiety^‡^ | -7.78 ± 9.47 | | 1.79 ± 8.62 | |  | 0.0028^**^ |
| Trait anxiety^‡^ | -6.39 ± 6.46 | | 3.42 ± 4.34 | |  | <0.0001^**^ |
| Hwa-Byung personality | -2.37 ± 4.37 | | 1.16 ± 5.33 | |  | 0.0321^*^ |
| Hwa-Byung symptom^§^ | -2.84 ± 7.07 | | 2.50 ± 6.15 | |  | 0.0196^*^ |
| WHOQOL-BREF^‡^ |  |  |  |  |  |  |
| Physical QOL | 1.62 ± 2.40 | | -0.54 ± 2.71 | |  | 0.0151^*^ |
| Psychological QOL | 1.52 ± 1.92 | | 0.00 ± 1.51 | |  | 0.0111^*^ |
| Social Relation | 0.96 ± 2.45 | | -0.91 ± 1.94 | |  | 0.0140^*^ |
| Environment | 1.44 ± 1.72 | | 0.13 ± 1.83 | |  | 0.0314^*^ |
| Data are mean±standard deviation. Mean represented as change calculated as 4weeks minus baseline. ** p < 0.01, * p < 0.05 (2-tailed independent two-sample t-test), ^†^ ANCOVA for PSS, ^‡^ The sample size of BQSRP is 18 because one participant in the BQSRP group did not complete the questionnaires for STAI, WHOQOL-BREF at baseline. ^§^The sample size of waiting is 18 because one participant in waiting group did not complete the questionnaire for *Hwa-Byung* symptom at baseline. | | | | | | |

**Table 4. Comparison of salivary cortisol hormone levels at baseline and change from baseline between groups (µg/dl)**

|  | | | | | | |
| --- | --- | --- | --- | --- | --- | --- |
|  | **BQSRP** | | **Wait-list** | |  | ***P*** |
|  | N | cortisol | N | cortisol |  |  |
| Baseline^†^ | 23 | 0.404±0.209 | 23 | 0.418±0.248 |  | 0.8378 |
| Change^‡^ | 17 | 0.015±0.141 | 17 | 0.004±0.162 |  | 0.8316 |
| Data are mean±standard deviation. ^†^The sample size of baseline is 23 per each group, the cortisol level of three samples was very low compared with the expected value. ^‡^The sample size of change is 17 per group, the cortisol levels of three samples (two at the baseline and one at the end point) were very low compared to the expected, one sample in the study completion could not be assessed due to the low amount of saliva, four samples (two in BQSRP, two in control group) were excluded for analysis. | | | | | | |
|  | | | | | | |
